# Reporting of the translation and cultural adaptation procedures of the Addenbrooke’s Cognitive Examination version III (ACE-III) and its predecessors: a systematic review

**DOI:** 10.1186/s12874-017-0413-6

**Published:** 2017-09-13

**Authors:** Nadine Mirza, Maria Panagioti, Muhammad Wali Waheed, Waquas Waheed

**Affiliations:** 1 Centre for Primary Care, The University of Manchester, Suite 6, 5th Floor, Williamson Building, Oxford Road, Manchester, M13 9PL UK; 2 The University of Leicester, Leicester, UK

**Keywords:** Cognitive assessment, cognitive impairment, cognitive screening test, dementia, diagnosis, primary care, systematic review, cultural adaptation, translation, language

## Abstract

**Background:**

The ACE-III, a gold standard for screening cognitive impairment, is restricted by language and culture, with no uniform set of guidelines for its adaptation. To develop guidelines a compilation of all the adaptation procedures undertaken by adapters of the ACE-III and its predecessors is needed.

**Methods:**

We searched EMBASE, Medline and PsychINFO and screened publications from a previous review. We included publications on adapted versions of the ACE-III and its predecessors, extracting translation and cultural adaptation procedures and assessing their quality.

**Results:**

We deemed 32 papers suitable for analysis. 7 translation steps were identified and we determined which items of the ACE-III are culturally dependent.

**Conclusions:**

This review lists all adaptations of the ACE, ACE-R and ACE-III, rates the reporting of their adaptation procedures and summarises adaptation procedures into steps that can be undertaken by adapters.

## Background

The Addenbrooke’s Cognitive Examination (ACE) [1] was developed in 1990 to act as a screening tool for detecting cognitive impairment while also incorporating the Mini Mental State Examination (MMSE) [2]. It was designed to assess the five cognitive domains attention, memory, verbal fluency, language and visuospatial abilities, as well as provide an overall indication of cognitive function. It was later updated in 2006 to account for cross cultural usage and improved sensitivity [3], resulting in the development of the Addenbrooke’s Cognitive Examination Revised (ACE-R) [4]. However, the ACE-R contained items that incorrectly indicated cognitive impairment and these were corrected in 2012, with sections of the MMSE removed due to copyright issues [5], resulting in the recent Addenbrooke’s Cognitive Examination Version III (ACE-III) [6].

The ACE-III is regarded as the gold standard for the screening and diagnostic accuracy of cognitive impairment [5]. It consists of 19 items, takes 15–20 min to administer and 5 min to score, scores being out of 100, and a higher score indicating healthier cognitive functioning [6, 7]. The ACE-III retains many of the items originally found in the ACE-R and the ACE, and is considered a comprehensive screening tool for cognitive impairment [5], comparing favourably to other standard neuropsychological tests [8]. It is not surprising therefore, that all three versions of the ACE [1, 4, 6] have been translated into various languages and are used widely across the globe. However, the ACE-III and its predecessors [1, 4, 6] have a key limitation. They have been designed for fluent English speakers aware of the cultural norms of the country where it was developed. Specific items such as verbal fluency and language require participants to read, speak, write and understand English [9–11].

Several translation and cultural adaptation procedures to produce suitable versions of psychometric instruments [1, 4, 6] are evidenced in the literature but there is an absence of a uniform set of guidelines to conduct the translation and adaptation of these instruments. The case of the ACE-III is not an exemption. At present, there is no formal guidance on the steps that should be undertaken in translating the ACE-III and its predecessors [1, 4, 6] into any given language and no official indication given as to which items of the ACE, ACE-R and ACE-III are culturally independent and which are culturally dependent and therefore may require a certain process to be appropriately adapted. The adaptation procedures for the ACE-III are fully dependent on replicating the procedures reported in previously published ACE adaptations. Yet, to the extent of our knowledge, there is no existing compilation of all the translation and culturally adaption procedures.

An important step for producing evidenced-based guidelines for the translation and cultural adaptation of the ACE-III is to conduct a systematic compilation of all the translation and culturally adaptation procedures undertaken by existing published versions of the ACE, ACE-R and ACE-III. This systematic review aims to meet this challenge. In this endeavour, we paid particular attention to recording the translation and cultural adaptation procedures reported by each publication because our primary focus was to identify the translation and cultural adaptation steps and processes and which items are deemed culturally dependent in which publication. To undertake this process as robustly as possible, we utilised two scales that assess the quality of reported translation and cultural adaptation, the Manchester Translation Reporting Questionnaire (MTRQ) and Manchester Cultural Adaptation Reporting Questionnaire (MCAR). These scales were applied to each publication to determine which report a procedure that can be successfully replicated by future cultural adapters of the ACE-III. We were able to produce a successful systematic review that lists all the existing translations and cultural adaptations of the ACE, ACE-R and ACE-III [1, 4, 6], and were able to rate all the reporting of their translation and cultural adaptation procedures on reliable scales that we have developed for public use. We were also able to extract all translation and cultural adaptation procedures from these publications, break them down and summarise them to allow for an understanding of what steps are most commonly undertaken by adapters. Such steps can be undertaken by future adapters when translating or culturally adapting the ACE-III for their purposes.

## Methods

Our methods followed a previously conducted systematic review and a meta-analysis of the Addenbrooke’s Cognitive Examination (ACE) and its revised version (ACE-R) [12, 13]. The guidelines on the reporting of systematic reviews in the PRISMA statement were followed [14].

### Search criteria

Due to the nature of the review the search was conducted using health care based electronic databases EMBASE, Medline and PsychINFO. The search terms were “addenbrooke's cognitive examination or ace-iii or screen* or test or instrument or measure or tool or diagnos*”, “dementia or Alzheimer* or cognitive”, and “sensitivity and specificity or accuracy or cut-off or receiver operator or ROC or Youden”. The search period was restricted from January 2013 to December 2015 as the first paper on the Addenbrooke’s Cognitive Examination Version III (ACE-III) was introduced in 2013 [6]. SCOPUS was also searched to locate publications that have cited the original paper by Hsieh et al. [6]. The search was run till 31st December 2016 to account for any new publications that could potentially meet our inclusion criteria but none were identified.

The searches were supplemented by screening all the included and excluded studies of Larner and Mitchell’s recent meta-analysis which examined the accuracy of ACE and ACE-R [13].

### Inclusion criteria

Publications that referred to translated and/or culturally adapted versions of the ACE, the ACE-R and the ACE-III, from English into any other language, and were the primary source of that version of the assessment, were included.

### Exclusion criteria

Publications that did not refer to a translated or culturally adapted version of the ACE, ACE-R or ACE-III were excluded.

### Study selection

The results of the searches in each database were exported to Endnote and duplicates were removed. Study selection was completed in two stages. First, the titles and abstracts of the identified studies were screened and subsequently the full-texts of relevant studies were accessed and further screened against the eligibility criteria. When the full texts were unavailable, the authors of the publications were contacted to provide additional information. A final attempt to obtain the publications was by contacting the respective journals and putting in a request. The full text of publications was read through by two authors individually (NM and MWW), who determined what language the ACE, ACE-R or ACE-III had been adapted to and extracted the section that described the translation and cultural adaptation process. The translation and cultural adaptation steps were identified in each extraction and separated.

### Analysis

The reported translation procedure of each publication was broken down into individual steps such that there was no overlap between two steps. The steps of all the publications were later merged and duplicates removed to create a list of all potential steps that could take place when translating the ACE, ACE-R and ACE-III. The reported cultural adaptation procedure of each publication was reviewed to identify which questions of the ACE, ACE-R and ACE-III were culturally dependent and how each question was appropriately adapted.

**Fig. 1:**
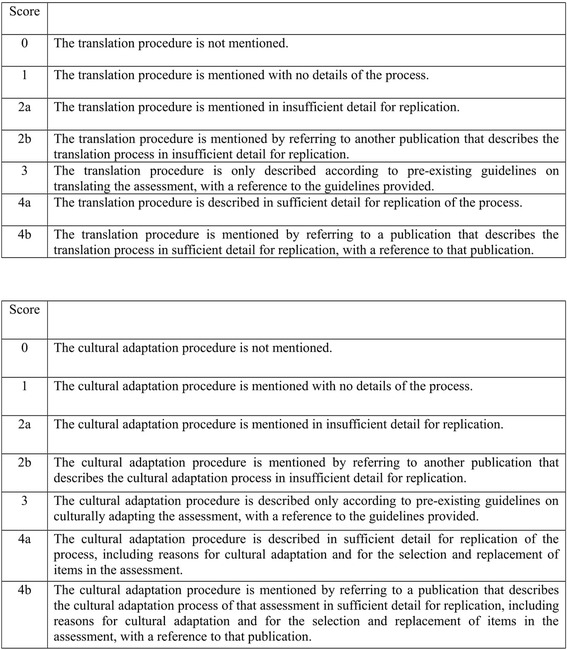
Manchester Translation Reporting Questionnaire (MTRQ) and Manchester Cultural Adaptation Reporting Questionnaire (MTRQ)

The quality of the reported translation and cultural adaptation procedures were assessed through the MTRQ and MCAR respectively. Both MTRQ and MCAR (See Fig. 1) are seven point rating scales that were developed at the Centre for Primary Care at the University of Manchester to quantify the overall quality of reported procedures undertaken in the translation or cultural adaptation of any assessment, in particular, neuropsychological assessments. In the development of these scales the quality of a described translation or cultural adaptation procedure is considered dependent on the extent to which it can be replicated successfully through the information it provides.

It is important to note that MTRQ and MCAR were designed to only account for reported translation and cultural adaptation procedures. Scores on the MTRQ and MCAR cannot assess the quality of the actual translation and cultural adaptation procedures that were potentially undertaken by adapters, only the manner in which they have reported it.

The reported translation and cultural adaptation procedures that were extracted by our two authors were also assessed by these authors independently, using the MTRQ and MCAR scales. At this stage an interrater reliability analysis, using the Kappa statistic, was performed to determine consistency among raters for both scales [15]. Following this, scores between the two authors that did not match were reviewed by a third author (WW) and a consensus was reached after discussion to determine the final score to be assigned to that publication.

## Results

### Search results

**Fig. 2:**
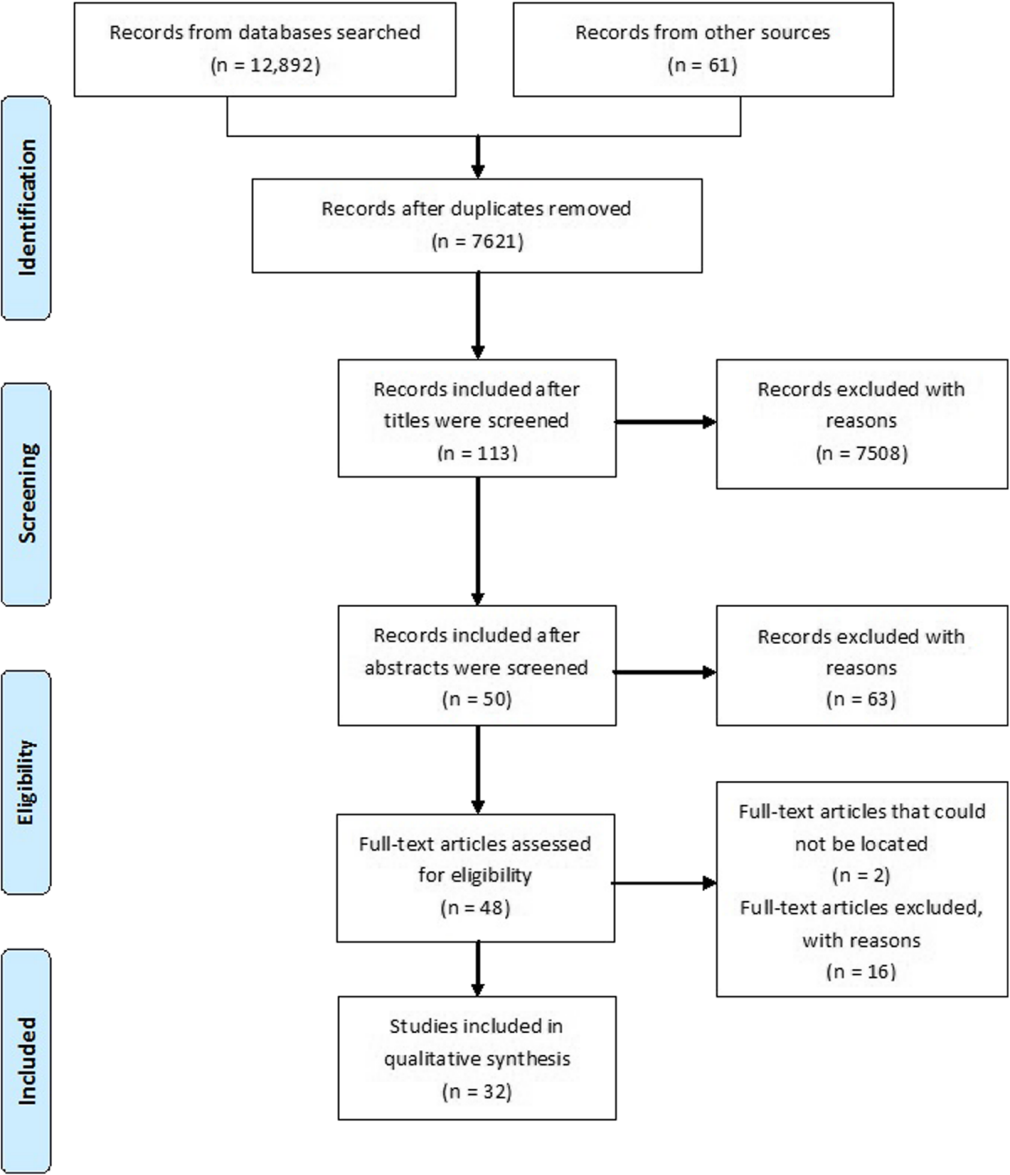
Flow diagram of the search process (PRISMA; Moher et al. [14])

Our search identified 113 publications on the ACE, ACE-R and ACE-III (See Fig. 2 for the PRISMA flow diagram). The abstracts of the publications were screened and 63 publications were excluded as they only focused on the original English versions of the ACE, ACE-R and ACE-III [1, 4, 6]. Full texts of the remaining 50 publications were searched for, including contacting authors and journals, of which two were not available to us. The remaining 48 publications were reviewed and 16 of these were excluded as they were not the primary papers for their respective non-English versions of the ACE, ACE-R and ACE-III. After final application of the exclusion criteria, 32 papers were deemed suitable for the analysis; 12 for ACE, 17 for ACE-R and 3 for ACE-III (See Table 1 for details on the papers that were analysed).

**Table 1:** All publications identified by our analysis with the reported translation steps undertaken

Authors	Year	ACE	Language	MTRQ	MCAR	Translation	Back Translation	Users in co-production	Expert Recommendations	Revisions based on step by step feedback	Involvement of original authors	Pilot Study
Alexopoulos et al. [16]	2006	ACE	German	4a	2a	X	X			X		
Bier et al. [30]	2004	ACE	French	4a	2a	X	X					X
Custodio et al. [22]	2012	ACE	Peruvian Spanish	0	2a							
Garcia-Caballero et al. [31]	2006	ACE	Spanish	2a	4a	X		X		X		X
Heo et al. [10]	2012	ACE	Korean	2a	4a	X			X			
Hummelová-Fanfrdlová et al. [32]	2009	ACE	Czech	4a	2a	X			X			
Mathuranath et al. [33]	2004	ACE	Malayalam	2a	4a	X	X					X
Newman. [28]	2006	ACE	Hebrew	2a	1	X						X
Pouretemad et al. [34]	2009	ACE	Persian	4a	4a	X	X		X			X
Sarasola et al. [25]	2004	ACE	Spanish	1	2a							
Stokholm et al. [29]	2009	ACE	Danish	4a	2a	X						
Yoshida et al. [35]	2011	ACE	Japanese	4a	4a	X	X					
Al-Salman. [9]	2013	ACE-R	Saudi Arabian	4a	4a	X	X	X	X	X		X
Alexopoulos et al. [36]	2010	ACE-R	German	4a	4a	X	X					
Bartoš et al. [37]	2011	ACE-R	Czech	2a	4a	X				X	X	
Bastide et al. [21]	2012	ACE-R	French	2b	2b	X						X
Carvalho et al. [38]	2007	ACE-R	Brazilian	4a	2a	X	X					
Dos Santos Kawata et al. [23]	2012	ACE-R	Japanese	1	4a							
Fang et al. [39]	2014	ACE-R	Chinese	2a	4a	X						
Ferreira et al. [27]	2012	ACE-R	Portuguese	2a	0	X					X	
Gondova et al. [18]	2012	ACE-R	Slovak	4a	4a	X	X	X	X	X	X	X
Konstantinopoulou et al. [40]	2011	ACE-R	Greek	4a	4a	X	X	X				X
Kwak et al. [24]	2010	ACE-R	Korean	1	1							
Margevičiūtė et al. [41]	2013	ACE-R	Lithuanian	4a	2a	X	X					
Munoz-Neira et al. [42]	2012	ACE-R	Chilean Spanish	2a	2a	X						X
Pigliautile et al. [19]	2011	ACE-R	Italian	4a	4a	X	X	X	X	X		X
Torralva et al. [43]	2011	ACE-R	Spanish	4a	2a	X	X					
Wong et al. [44]	2013	ACE-R	Cantonese	2a	4a	X			X			
Yoshida et al. [20]	2012	ACE-R	Japanese	2a	4a	X	X				X	X
Machado et al. [26]	2015	ACE-III	Portuguese	4a	0	X	X			X		
Matias-Guiu et al. [11]	2015	ACE-III	Spanish	4a	4a	X				X		X
Qassem et al. [17]	2014	ACE-III	Egyptian Arabic	4a	2a	X	X			X		X

### Quality of translation and cultural adaptation reporting

The quality of the reported translation and cultural adaptation procedures were rated on the MTRQ and MCAR scales respectively (See Table 1 for the MTRQ and MCAR scores of the papers that were accessible to us in full text). The interrater reliability for the raters regarding the MTRQ was found to be Kappa = 0.242 (*p* > 0.001), 95% CI (0.426, 0.058). The interrater reliability for the raters regarding the MCAR was found to be Kappa = 0.581 (*p* < 0.001), 95% CI (0.797, 0.365) [15].

### Reported translation and cultural adaptation

This review identified seven individual steps that can be undertaken when translating the ACE, ACE-R and ACE-III:

i. Translation: Direct translation, without any form of cultural adaptation, from English into the target language, often with the assistance of a native or fluent speaker of the language or an official translator. Eg. “Independently of one another, two members of our group translated the ACE into German” [16].
ii. Back Translation: Creating a retroversion of the initial translation, from the target language back to English, often with the assistance of a native or fluent speaker of the language or an official translator. Eg. “First, the questionnaire was translated into Arabic, and then back translated into English. The process was then repeated until a consensus was reached about the optimal translation” [17].
iii. Users in Coproduction: Potential or future users of the assessment, such as those from a target population, including native and fluent speakers of the language, providing feedback or information in any way that influences the development of the translated assessment. Eg. “There were […] cognitively healthy participants of age range 60–70 who were tested with Slovak ACE-R […]. Afterwards the participants were asked for a feedback especially on the questions where more possibilities were considered to be used in the final version” [18].
iv. Expert Recommendations: Experts on translation, the target languages, or subject matters related to the assessment providing feedback or information in any way that influences the development of the translated assessment. Eg. “A review committee composed by two psychologists and two geriatricians chose the final version of the Italian ACE-R” [19].
v. Revisions based on step-by-step feedback: Constant and continuous revisions of the translated assessment developed whenever a change or suggestion is proposed and approved. Eg. “At different points of this process, members of the original team and Spanish speakers, suggested modifications [19]”.
vi. Involvement of the original authors: Authors of the original assessment providing feedback or information in any way that influences the development of the translated assessment. Eg. “We translated and modified ACE-R with advice from the authors of the original version” [20].
vii.  Pilot Study: Administering translated versions of the assessment to assess its feasibility and acceptability amongst potential users. Eg. “The ACE plus 1 of the 3 new ACE-R versions were administered to patients who were examined by one of the authors (J.-C.B.) at the ERASME Hospital Memory Clinic” [21].

Out of the 32 papers only one, Custodio et al. [22] did not mention a translation process occurring at all, resulting in a score of 0 on the MTRQ. 3 of the papers (Dos Santos et al. [23], Kwak et al. [24], and Sarasola et al. [25]) mentioned that a translation process took place but not in enough detail to determine any individual steps or elaborate on the process. This is reflected in their receiving a score of 1 on the MTRQ.

Table 1 also shows the translation steps reported by the remaining 28 papers, indicating the frequency with which individual translation steps were reportedly undertaken. Only one paper, Gondova et al. [18], reported undertaking all the potential steps for translating that this review has identified. The remaining 27 papers reportedly undertook translation steps in various combinations; Translation was undertaken by all the papers, 16 papers undertook back translation, 5 papers reported users in coproduction, 7 papers approached experts for recommendations, 9 papers made revisions based on step-by-step feedback, 4 papers involved original authors and 14 papers conducted pilot studies.

Out of the 32 Machado et al. [26] and Ferreira et al. [27] did not mention a cultural adaptation process at all, resulting in a score of 0 on the MCAR. 2 papers, Kwak et al. [24] and Newman [28], mentioned that a cultural adaptation process took place but did not describe it, therefore receiving a score of 1 on the MCAR.

Table 2 shows which items of the ACE, ACE-R and ACE-III were reportedly culturally adapted by which of the remaining 28 papers, indicating the frequency with which individual items were culturally adapted across publications. Items 3: Attention and Concentration, 5b: Fluency - Animals, 15a: Visuospatial Abilities - Infinity Diagram, 15b: Visuospatial Abilities - Wire Cube and 16: Visuospatial Abilities were not culturally adapted across any of the papers as they only required direct translation. The remaining items were culturally adapted by various numbers of publications; Items 8: Language – Comprehension and 9: Language – Writing were culturally adapted in 1 paper, items 1: Attention - Orientation, 2: Attention - Registration, 4: Memory – Recall and 15c: Visuospatial Abilities - Clock in 2 papers, item 17: Visuospatial Abilities in 6 papers, items 5a: Fluency - Letters, 12: Language – Naming and 13: Language - Comprehension in 10 papers, item 11: Language Repetition in 13 papers, item 14: Language – Reading in 20 papers, item 10: Language - Repetition in 21 papers, item 7: Memory – Retrograde in 23 papers, item 18: Memory - Recall in 26 papers and item 6: Memory - Anterograde was culturally adapted in all 28 papers. Item 19: Memory - Recognition does not exist in the ACE but was culturally adapted in 16 papers out of the 17 ACE-R and ACE-III papers.

**Table 2:** The frequency of cultural adaptation per item of the ACE-III

Papers	1	2	3	4	5a	5b	6	7	8	9	10	11	12	13	14	15a	15b	15c	16	17	I8	19
Alexopoulos et al. [16]							X	X			X	X			X						X	N/A
Bier et al. [30]							X	X			X	X			X						X	N/A
Custodio et al. [22]							X	X			X	X						X			X	N/A
Garcia-Caballero et al. [31]							X	X			X	X			X						X	N/A
Heo et al. [10]					X		X	X			X	X			X						X	N/A
Hummelová-Fanfrdlová et al. [32]																						N/A
Mathuranath et al. [33]	X				X		X	X			X	X	X		X						X	N/A
Pouretemad et al. [34]					X		X	X			X	X	X	X	X						X	N/A
Sarasola et al. [25]							X	X			X	X			X						X	N/A
Stokholm et al. [29]							X														X	N/A
Yoshida et al. [35]					X		X				X	X			X						X	N/A
Alexopoulos et al. [36]							X	X			X										X	X
Al-Salman [9]					X		X	X			X		X	X	X					X	X	X
Bartoš et al. [37]	X						X						X									
Bastide et al. [21]							X	X			X										X	X
Carvalho et al. [38]							X	X					X	X	X						X	X
Dos Santos Kawata et al. [23]							X	X			X		X	X	X					X	X	X
Fang et al. [39]		X		X	X		X	X			X			X							X	X
Gondova et al. [18]							X	X			X				X						X	X
Konstantinopoulou et al. [40]							X	X			X		X		X						X	X
Margevičiūtė et al. [41]							X								X						X	X
Munoz-Neira et al. [42]							X	X													X	X
Pigliautile et al. [19]					X		X	X							X			X		X	X	X
Torralva et al. [43]							X		X	X	X	X	X	X	X						X	X
Wong et al. [44]					X		X	X			X			X	X					X	X	X
Yoshida et al. [20]					X		X	X			X	X	X	X	X					X	X	X
Matias-Guiu et al. [11]		X		X			X	X			X	X		X	X						X	X
Qassem et al. [17]					X		X	X			X	X	X	X	X					X	X	X

The frequency of cultural adaptation:

1: Attention - Orientation. 2: Attention - Registration. 3: Attention and Concentration. 4: Memory - Recall. 5a: Fluency - Letters

5b: Fluency - Animals. 6: Memory - Anterograde. 7: Memory - Retrograde. 8: Language – Comprehension. 9: Language - Writing

10: Language - Repetition. 11: Language - Repetition. 12: Language - Naming. 13: Language - Comprehension 14: Language - Reading

15a: Visuospatial Abilities - Infinity Diagram. 15b: Visuospatial Abilities - Wire Cube. 15c: Visuospatial Abilities - Clock. 16: Visuospatial Abilities. 17: Visuospatial Abilities. 18: Memory - Recall. 19: Memory - Recognition

Out of the 32 papers that mentioned translation and cultural adaptation procedures in some level of detail, 16 papers elaborated further on their processes in terms of individuals involved. Table 3 further elaborates on who these individuals were and which papers reported on their involvement; 9 papers mentioned bilingual experts and researchers, 6 papers mentioned psychiatrists and psychologist, 3 papers mentioned accredited translators, 3 papers mentions experts in linguistics, 2 papers mentioned physicians and neurologists, 1 paper mentioned geriatricians, 1 paper mentioned speech therapists, 1 paper mentioned care givers and 1 paper mentioned test administrators.

**Table 3:** Individuals reportedly involved in translation and cultural adaptation

Papers	Psychiatrist/Psychologists	Physicians/Neurologists	Geriatricians	Bilingual experts/researchers	Accredited translators	Experts in linguistics	Speech therapists	Care Givers	Test Administrators
Alexopoulos et al. [16]				x					
Bier et al. [30]				x					
Heo et al. [12]		x							
Hummelova-Fanfrdlova et al. [32]				x					
Pouretemad et al. [34]	x				x				
Yoshida et al. [35]				x					
Al-Salman [9]	x			x	x	x			
Alexopoulos et al. [36]				x					
Bartoš et al. [37]	x	x					x	x	x
Gondova et al. [18]	x			x		x			
Konstantinopoulou et al. [40]				x					
Margevičiūtė et al. [41]					x				
Pigliautile et al. [19]	x		x						
Wong et al. [44]						x			
Yoshida et al. [20]				x					
Machado et al. [26]	x								

## Discussion and conclusions

Conclusively, our review summarises the reported translation and cultural adaptation procedures of the stated 32 publications, which can guide those who may want to translate and adapt the ACE-III in future. However, we were also able to successfully identify which of these publications reported procedures in sufficient detail for potential adapters to be able to replicate the process (See Table 1) through utilisation of the MTRQ and the MCAR. Inter rater reliability of the MTRQ was found to be fair and for the MCAR was found to be moderate [15], however, comparisons of the scores with a third author as a mediator and better understanding of the scales deemed the scores we assigned in this review as suitable ratings for the ACE, ACE-R and ACE-III publications. We can also determine from our scoring of the ACE, ACE-R and ACE-III publications, that the MTRQ and MCAR scores are not dependent on one another; a publication may score high on the MTRQ due to a high quality of reporting of the translation procedure and low on the MCAR due to a low quality of reporting of the cultural adaptation procedure and vice versa.

However, it is important to acknowledge that the MTRQ and the MCAR cannot assess the quality of translation or cultural adaptation of an assessment. As it focuses only on the *reporting* of the translation and adaptation procedure, not of the actual assessment itself, it can only determine if the quality of reporting is of a high standard. A publication may report the translation and cultural adaptation procedure in sufficient detail for it to be replicated, yet this may still result in the production of an inappropriate or poorly adapted assessment. Or, as is the case with our review, we accounted for translation steps and cultural adaptation processes that were reported across the 32 full text publications we had access to, not necessarily whether cultural adaptation or translation took place. Adapters of each non-English version of the ACE, ACE-R and ACE-III may have translated the assessment and culturally adapted questions and not mentioned it in their publications, resulting in a score of 1 or 0.

Despite this, having access to scales that can assess the quality of reporting of the translation and cultural adaptation procedures within publications provides future adapters with a crucial starting point to determine which publications’ procedures they should consider replicating, particularly when, as in the case of the ACE-III and its predecessors, there are no formal guidelines or instructions.

Overall, our review has provided an existing summation of all publications that introduce a translated and culturally adapted version of the ACE, ACE-R and ACE-III. A complete list such as this is a point of referral for designing future translation and cultural adaptations of the ACE-III and to locate any currently existing versions. However, we were not only able to identify all the existing publications, but also the translation and cultural adaptation procedures reported within these publications in their entirety.

A full list of independent translation steps were identified and defined which can be undertaken by future adapters and the frequency of the occurrence of each of these steps across the publications that reported translation steps, both in sufficient and insufficient detail, has been tabulated (See Table 1). We can see that direct translation was the most common step, undertaken by all the publications, followed by back translation, pilot studies, revisions based on step by step feedback, expert recommendations, users in coproduction and lastly, the involvement of original authors, which was reportedly only undertaken by four publications. The frequency of the translations steps across publications will allow future adapters to make judgement calls regarding which steps they may endeavour to undertake as this review highlights the translation steps that previous adapters sought to follow.

We were also able to identify which items were considered culturally dependent by which publications and which items were most likely to be culturally adapted. Table 2 successfully shows the frequency of reported cultural adaptation taking place across any non-English version of the ACE, ACE-R or ACE-III and for any given item of the ACE, ACE-R or ACE-III. For example, item 6: Memory – Anterograde has been culturally adapted in all 28 of the publications whereas item 15c: Visuospatial Abilities – Clock has only been reportedly culturally adapted in two publications. In the same way, Qassem et al. [17] and Yoshida et al. [20] were reportedly the most culturally adapted publications while Stokholm et al. [29] was the least. This will indicate to future adapters which items were prioritised for cultural adaptation by previous adapters.

The knowledge of which individuals were involved in the translation and cultural adaptation of each version were also extracted and described in Table 3. This provides information regarding who these individuals were most likely to be. This is particularly important as accurate replication of the reported translation and cultural adaptation procedures can only occur with existing knowledge of who was involved in adapting the assessment. Bilingual experts were most commonly involved, having been mentioned in 9 publications, followed by psychologists and psychiatrists, accredited translators, linguistic experts and physicians and neurologists. Geriatricians, speech therapists, care givers and test administrators were only involved in one publication each.

Overall, we were able to produce a successful systematic review that lists all the existing translations and cultural adaptations of the ACE, ACE-R and ACE-III [1, 4, 6], and were able to rate all the reporting of their translation and cultural adaptation procedures on reliable scales that we have developed for public use. We were also able to extract all translation and cultural adaptation procedures from these publications, break them down and summarise them to allow for an understanding of what steps are most commonly undertaken by adapters. Such steps can be undertaken by future adapters when translating or culturally adapting the ACE-III for their purposes.

## References

[1] Mathuranath PS, Nestor PJ, Berrios GE, Rakowicz W, Hodges JR (2000). A brief cognitive test battery to differentiate Alzheimer’s disease and frontotemporal dementia. Neurology.

[2] Folstein MF, Folstein SE, McHugh PR (1975). Mini-mental state: A practical method for grading the cognitive state of patients for the clinician. J Psychiatr Res.

[3] Nooone P (2015). Questionnaire review: Adenbrooke’s cognitive examination III. Occup Med (Oxf).

[4] Mioshi E, Dawson K, Mitchell J, Arnold R, Hodges JR (2006). The Addenbrooke’s cognitive examination revised (ACE-R): a brief cognitive test battery for dementia screening. Int J Geriatr Psychiatry.

[5] Cheung G, Clugston A, Croucher M, Malone D, Mau E, Sims A, Gee S (2015). Performance of three cognitive screening tools in a sample of older New Zealanders. Int Psychogeriatr.

[6] Hsieh S, Schubert S, Hoon C, Mioshi E, Hodges J (2013). Validation of the Addenbrooke’s cognitive examination III in frontotemporal dementia and Alzheimer’s disease. Dement Geriatr Cogn Disord.

[7] NeuRA 2012, www.neura.edu.au/frontier/research).

[8] Velayudhan L, Ryu S, Raczek M, Philpot M, Lindesay J, Critchfield M, Livingston G (2014). Review of brief cognitive tests for patients with suspected dementia. Int Psychogeriatr.

[9] Al-Salman ASA. The Saudi Arabian adaptation of the Addenbrooke’s cognitive examination revised (ACE-R). Glasgow: University Glasgow. 2013. p. 40–65.

[10] Heo JH, Lee KM, Park TH, Ahn JY, Kim MK (2012). Validation of the Korean Addenbrooke’s cognitive examination for diagnosing Alzheimer’s dementia and mild cognitive impairment in the Korean elderly. Appl Neuropsychol.

[11] Matias-Guiu JA, Fernández de Bobadill R, Escudero G, Pérez-Pérez J, Cortés A, Morenas-Rodríguez E, Valles-Salgado M, Moreno-Ramos T, Kulisevsky J, Matías-Guiu J. Validation of the Spanish version of Addenbrooke’s cognitive examination III for diagnosing dementia. Neurologia. 30(9):545–51. 10.1016/j.nrl.2014.05.004 25002342

[12] Crawford S, Whitnall L, Robertson J, Evans JJ (2012). A systematic review of the accuracy and clinical utility of the Addenbrooke’s cognitive examination and the Addenbrooke’s cognitive examination revised in the diagnosis of dementia. Int J Geriatr Psychiatry.

[13] Larner AJ, Mitchell AJ (2014). A meta-analysis of the accuracy of the Addenbrooke’s cognitive examination (ACE) and the Addenbrooke’s cognitive examination revised (ACE-R) in the detection of dementia. Int Psychogeriatr.

[14] Moher D, Liberati A, Tetzlaff J, Altman DG, The PRISMA Group. Preferred reporting items for Systematic Reviews and Meta-Analyses: The PRISMA Statement. PLoS Med. 6(7):e1000097. https://doi.org/10.1371/journal.pmed.1000097. 10.1371/journal.pmed.1000097 PMC2707599 19621072

[15] Landis JR, Koch GG (1977). The measurement of observer agreement for categorical data. Biometrics.

[16] Alexopoulos P, Greim B, Nadler K, Martens U, Krecklow B, Domes G, Herpertz S, Kurz A (2006). Validation of the Addenbrooke’s cognitive examination for detecting early Alzheimer’s disease and mild vascular dementia in a German population. Dement Geriatr Cogn Disord.

[17] Qassem T, Khater MS, Emara T, Tawfik HM, Rasheedy D, Mohammedin AS, Tolba MF, Aziz KA: Translation and cross cultural adaptation of the Addenbrooke’s cognitive examination III into Egyptian Arabic. Royal College of Psychiatrists International Congress. 2014.

[18] Gondova: Translation and Cultural Adaptation of the Addenbrooke’s Cognitive Examination – Revised (ACE-R) for the Slovak Population. The University of Edinburgh. 2012; 14–16.

[19] Pigliautile M, Ricci M, Mioshi E, Ercolani S, Mangialache F, Monastero R, Croce MF, Federici S, Mecocci P (2011). Validation study of the Italian Addenbrooke’s Cognitive Examination Revised in a young-old and old-old population. Dement Geriatr Cogn Disord.

[20] Yoshida H, Terada S, Honda H, Kishimoto Y, Takeda N, Oshima E, Hirayama K, Yokota O, Uchitomi Y (2012). Validation of the revised Addenbrooke’s cognitive examination (ACE-R) for detecting mild cognitive impairment and dementia in a Japanese population. Int Psychogeriatr.

[21] Bastide L, De Breucker S, Van den Berge M, Fery P, Pepersack T, Bier JC (2012). The Addenbrooke’s Cognitive Examination Revised is as effective as the original to detect dementia in a French-speaking population. Dement Geriatr Cogn Disord.

[22] Custodio N, Lira D, Montesinos R, Gleichgerrcht E, Manes F (2012). Utilidad del Addenbrooke’s cognitive examination versión en español en pacientes peruanos con enfermedad de Alzheimer y demencia frontotemporal. Rev Arg de Psiquiat.

[23] Dos Santos Kawata KH, Hashimoto R, Nishio Y, Hayashi A, Ogawa N, Kanno S, Hiraoka K, Yokoi K, Iizuka O, Mori E (2012). A validation study of the Japanese version of the Addenbrooke’s cognitive examination revised. Dement Geriatr Cogn Disord.

[24] Kwak YT, Yang Y, Kim GW (2010). Korean Addenbrooke’s cognitive examination revised (K-ACER) for differential diagnosis of Alzheimer’s disease and subcortical ischemic vascular dementia. Geriatr Gerontol Int.

[25] Sarasola D, Calcagno MDL, Sabe L, Caballero A, Manes F (2004). Utilidad del Addenbrooke’s cognitive examination en Español para el diagnóstico de demencia y para la diferenciación entre la enfermedad de Alzheimer y la demencia frontotemporal. Rev Argent Neuropsicol.

[26] Machado A, Baeta E, Pimentel P, Peixoto B (2015). Psychometric and normative indicators of the Portuguese version of the Addenbrooke’s cognitive examination III: Preliminary study on a sample of healthy subjects. Acta Neuropsychol.

[27] Ferreira IS, Simoes MR, Maroco J (2012). The Addenbrooke’s cognitive examination revised as a potential screening test for elderly drivers. Accid Anal Prev.

[28] Newman JP (2005). Brief assessment of cognitive mental status in Hebrew: Addenbrooke’s cognitive examination. Isr Med Assoc J.

[29] Stokholm J, Vogel A, Johannsen P, Waldemar G (2009). Validation of the Danish Addenbrooke’s cognitive examination as a screening test in a memory clinic. Dement Geriatr Cogn Disord.

[30] Bier JC, Ventura M, Donckels V, Van Eyll E, Claes T, Slama H, Fery P, Vokaer M, Pandolfo M (2004). Is the Addenbrooke’s Cognitive Examination effective to detect frontotemporal dementia? J. Neurol.

[31] Garcia-Caballero A, Garcia-Lado I, Gonzalez-Hermida J, Recimil MJ, Area R, Manes F, Lamas S, Berrios GE (2006). Validation of the Spanish version of the Addenbrooke’s Cognitive Examination in a rural community in Spain. Int J Geriatr Psychiatry.

[32] Hummelová-Fanfrdlová Z, Rektorová I, Sheardová K, Bartos A, Línek V (2009). Ceska adaptace, Addenbrookskeho kognitivniho testu. Cesk Psychol.

[33] Mathuranath PS, Hodges JR, Mathew R, Cherian PJ, George A, Bak TH (2004). Adaptation of the ACE for a Malayalam speaking population in southern India. Int J Geriatr Psychiatry.

[34] Pouretemad HR, Khatibi A, Ganjavi A, Shams J, Zarei M (2009). Validation of Addenbrooke’s cognitive examination (ACE) in a Persian-speaking population. Dement Geriatr Cogn Disord.

[35] Yoshida H, Terada S, Honda H, Ata T, Takeda N, Kishimoto Y, Etsuko O, Ishihara T, Kuroda S (2011). Validation of Addenbrooke’s cognitive examination for detecting early dementia in a Japanese population. Psychiatry Res.

[36] Alexopoulos P, Ebert A, Richter-Schmidinger T, Scholl E, Natale B, Aguilar CA, Gourzis P, Weih M, Perneczky R, Diehl-Schmid J, Kneib T, Forstl H, Kurz A, Danek A, Kornhuber J (2010). Validation of the German revised Addenbrooke’s cognitive examination for detecting mild cognitive impairment, mild dementia in Alzheimer’s disease and frontotemporal lobar degeneration. Dement Geriatr Cogn Disord.

[37] Bartoš A, Raisová M, Kopeček M (2011). Novelizace české verze Addenbrookského kognitivního testu (ACE-CZ). Cesk Neurol Neurochir.

[38] Carvalho VA, Barbosa MT, Caramelli P (2007). Brazilian adaptation of the Addenbrooke’s Cognitive Examination Revised. Neuropsychologia.

[39] Fang R, Wang G, Huang Y, Zhuang JP, Tang HD, Wang Y, Deng YL, Xu W, Chen SD, Ren RJ (2014). Validation of the Chinese version of Addenbrooke’s cognitive examination-revised for screening mild Alzheimer’s disease and mild cognitive impairment. Dement Geriatr Cogn Disord.

[40] Konstantinopoulou E, Kosmidis MH, Ioannidis P, Kiosseoglou G, Karacostas D, Taskos N (2011). Adaptation of Addenbrooke’s Cognitive Examination-Revised for the Greek population. Eur J Neurol.

[41] Margevičiūtė R, Bagdonas A, Butkus K, Kuzmickiene J, Vaitkevicius A, Kaubrys GF, Bak TH (2013). Adenbruko kognityvinio tyrimo metodikos – taisytos adaptacija lietuviškai kalbantiems gyventojams (ACE-RLT). Neurologijos seminarai.

[42] Munoz-Neira C, Henríquez CF, Ihnen JJ, Sánchez CM, Flores MP, Slachevsky CA (2012). Psychometric properties and diagnostic usefulness of the Addenbrooke's Cognitive Examination-Revised in a Chilean elderly sample. Rev Med Chil.

[43] Torralva T, Roca M, Gleichgerrcht E, Bonifacio A, Raimondi C, Manes F (2011). Validation of the Spanish version of the Addenbrooke’s Cognitive Examination-Revised (ACE-R). Neurologia.

[44] Wong L, Chan C, Leung J, Yung CY, Wu KK, Cheung SYY, Lam CLM (2013). A validation study of the Chinese-Cantonese Addenbrooke’s Cognitive Examination Revised (C-ACER). Neuropsychiatr Dis Treat.

